# Adhesion of human gingival fibroblasts/*Streptococcus mitis* co-culture on the nanocomposite system Chitlac-nAg

**DOI:** 10.1007/s10856-016-5701-x

**Published:** 2016-03-12

**Authors:** Amelia Cataldi, Marialucia Gallorini, Mara Di Giulio, Simone Guarnieri, Maria Addolorata Mariggiò, Tonino Traini, Roberta Di Pietro, Luigina Cellini, Eleonora Marsich, Silvia Sancilio

**Affiliations:** Department of Pharmacy, G. d’Annunzio University, Chieti-Pescara, Via dei Vestini 31, 66100 Chieti Scalo, CH Italy; Center for Aging Science (Ce.S.I.), G. d’Annunzio University Foundation, Chieti, Italy; Department of Neuroscience, Imaging and Clinical Sciences, G. d’Annunzio University, Chieti-Pescara, Chieti, Italy; Department of Medical, Oral and Biotechnological Sciences, G. d’Annunzio University, Chieti-Pescara, Chieti, Italy; Department of Medicine and Ageing Sciences, G. d’Annunzio University, Chieti-Pescara, Chieti, Italy; Department of Medical, Surgical and Health Sciences, University of Trieste, Trieste, Italy

## Abstract

Composite materials are increasingly used as dental restoration. In the field of biomaterials, infections remain the main reason of dental devices failure. Silver, in the form of nanoparticles (AgNPs), ions and salt, well known for its antimicrobial properties, is used in several medical applications in order to avoid bacterial infection. To reduce both bacterial adhesion to dental devices and cytotoxicity against eukaryotic cells, we coated BisGMA/TEGDMA methacrylic thermosets with a new material, Chitlac-nAg, formed by stabilized AgNPs with a polyelectrolyte solution containing Chitlac. Here we analyzed the proliferative and adhesive ability of human gingival fibroblasts (HGFs) on BisGMA/TEGDMA thermosets uncoated and coated with AgNPs in a coculture model system with *Streptococcus mitis*. After 48 h, HGFs well adhered onto both surfaces, while *S. mitis* cytotoxic response was higher in the presence of AgNPs coated thermosets. After 24 h thermosets coated with Chitlac as well as those coated with Chitlac-nAg exerted a minimal cytotoxic effect on HGFs, while after 48 h LDH release raised up to 20 %. Moreover the presence of *S. mitis* reduced this release mainly when HGFs adhered to Chitlac-nAg coated thermosets. The reduced secretion of collagen type I was significant in the presence of both surfaces with the co-culture system even more when saliva is added. Integrin β1 localized closely to cell membranes onto Chitlac-nAg thermosets and PKCα translocated into nuclei. These data confirm that Chitlac-nAg have a promising utilization in the field of restorative dentistry exerting their antimicrobial activity due to AgNPs without cytotoxicity for eukaryotic cells.

## Introduction

Composite materials are increasingly used in restorative dentistry, but in the broad field of biomaterials, infections remain the main reason of dental devices failure [1]. The surface of the oral cavity is exposed to a broad variety of microorganisms capable of forming biofilms [2], not only on oral mucosa and teeth surfaces, but also on the components used for dental restoration [3, 4]. It is well known that resin-based composites are susceptible to microbial colonization and to biofilm formation in the oral cavity. Oral microbial biofilms produce acid and are involved in the occurrence of periodontitis and dental caries [5]. Recently, in order to avoid the proliferation and the bacterial adhesion on material surface, materials with antimicrobial properties have been set up [6, 7]. Silver, in the form of nanoparticles, ions and salt, well known for its antimicrobial properties, is used in several medical and general applications in order to avoid bacterial infection [8, 9]. Silver ions and nanoparticles are capable to destroy the bacterial cell wall by means of the reaction with electron donor groups. Silver acts on sulfhydryl groups on trans-membrane and outer-membrane proteins, including proteins of the electron transport chain, protruding into the extracellular portion of the membrane [10], while in eukaryotic cells lacking these extracellular binding sites, the potential cytotoxic effect could be due to silver internalization [11]. This nanoparticles diffusion into the cells can lead to their death interfering with several metabolic pathways [10]. During the preparation, to prevent silver nanoparticles aggregation, which leads to the loss of the antimicrobial activity, associated with the nanoscale [12], the polyelectrolyte Chitlac (lactose-modified chitosan) at low concentrations is used to stabilize silver nanoparticles colloidal solutions [11]. Immobilizing silver nanoparticles into a biocompatible polymeric film in order to allow nanoparticles interaction with bacterial membrane without affect eukaryotic cells is necessary to minimize the interaction between eukaryotic cells and silver nanoparticles [11]. In this work the substrate material is a thermoset based on Bisphenol A glycidylmethacrylate (BisGMA) and triethylenglicol dimethacrylate (TEGDMA), widely used for dental devices [13]. Chitlac-nAg coated methacrylic thermosets have been already tested in order to assay the interaction of the coating either with bacteria either with eukaryotic cells [11]. It cannot be forgotten that oral cavity environment consists of eukaryotic gingival epithelial and fibroblasts cells, human oral microbiota and saliva. The oral surface is kept wet by saliva [14]. This fluid is essential for the maintenance of the oral ecosystems by ensuring water, nutrients, adherence and antimicrobial factors. Saliva contains 99 % water and glycoproteins (for example mucins), proteins, hormones, vitamins, urea and several ions. The role of mucins in bacterial adherence is complex. When salivary glycoproteins are adsorbed on solid surface, they may bind to bacteria and promote bacterial adherence [15]. Instead, some of these glycoproteins, when free in saliva, may prevent bacterial colonization by binding to their adhesion proteins or by agglutinating bacteria in saliva [16]. Within the oral microbiota, Streptococci constitute the main population of bacteria, able to foster tight associations with host epithelial tissue and resulting in positive effects on healthy oral status [17]. In particular, *Streptococcus mitis* represents one of the first colonizers of hard (teeth) as well as mucosal surfaces [18]. Thus, a eukaryotic/prokaryotic co-culture model, a first step to try to mimic “in vitro” the “in vivo” oral cavity environment, here was analyzed as a biological model [17, 19, 20].

Integrin α5β1, a heterodimeric transmembrane protein able to bind extracellular ligands as well as intracellular proteins involved in cell signaling, represents the main bidirectional receptor connecting the cytoskeleton and the extracellular matrix (ECM) in HGFs [21]. Numerous data suggest that the binding of various bacterial modulins to their cognate receptors available on different eukaryotic cells leads to the release of inflammatory cytokines playing an important role in periodontal diseases [22]. In addition, previous studies suggest that integrin β1 can also trigger PKCα isoenzyme in response to resin composite monomers released from dental materials [23].

The aim of this study was to investigate the proliferative and adhesive behaviour of HGFs on BisGMA/TEGDMA thermosets uncoated and coated with AgNPs in our co-culture model. Then the molecular mechanisms driving such processes were studied in order to clarify biological reactions that can occur between biomaterials, host tissue and microbial environment.

## Materials and methods

### Preparation of BisGMA/TEGDMA thermosets (uncoated samples) and Chitlac-nAg coating on the BisGMA/TEGDMA thermosets

BisGMA (70 % w/w) and TEGDMA (30 % w/w) were mixed under vigorous stirring at 37 °C. Camphorquinone (CQ, 0.7 % w/w) and 2-dimethylamino ethylmethacrylate (DMAEMA, 0.7 % w/w) were added and the solution was protected from light and degassed for 12 h in vacuum oven at 40 °C. The solution was poured in Teflon mold (diameter 14 mm, height 2.5 mm) and the wells were covered with a polyethylene terephthalate (PET) film. The polymerization was light initiated with a hand cure light device (Optilux 501, *λ*: 400–505 nm, light power: 850 mW/cm^2^) for 20 s. The postcuring was performed with a Photopol IR/UV Plus oven (Dentalfarm, Torino, Italy) equipped with eight lamps and two spots operating in the wavelength range 320–550 nm following the procedure: 20 min in light oven (eight lamps), 20 min in light oven (eight lamps) on a rotating plate, 60 min in light oven (eight lamps) under vacuum, and 7 min in light oven (eight lamps plus two spots). The thermosets were then sandpaper polished (granulometry: 1200) [11].

Chitlac (1-deoxylactit-1-yl chitosan, CAS registry number 85941-43-1) sample was prepared according to the procedure elsewhere reported [24]. BisGMA/TEGDMA thermosets were prepared and coated with Chitlac–nAg, as already reported [11].

### Culture of human gingival fibroblasts

Human gingival fibroblasts (HGFs) were obtained at the Dental Clinic of the Department of Medical, Oral and Biotechnological Sciences of the University G. d’Annunzio. The tissue fragments were treated as previously described [25]. Signed informed consent was obtained from the donors. None of the authors participated to the sample collection and samples were anonymized before the authors received them. Cells were used from passage 5 up to 12.

### Bacterial strains and growth condition, saliva collection and co-culture set up

The clinical strain *S. mitis* DS12 from a saliva sample was used in the present study. The strain was cultured as previously described [17].

Pooled unstimulated saliva was obtained from laboratory healthy staff (after no drinking or eating for two hours) by spitting method in polypropylene tube. The saliva was treated as previously described [25].

The co-culture assay was performed as reported elsewhere [25]. The experimental design was carried out for at least three independent experiments.

The different experimental conditions are the following: human gingival fibroblasts (H), HGFs on Chitlac coated thermoset (ThH), HGFs on Chitlac-nAg coated thermoset (AgH), *S. mitis* on Chitlac coated thermoset (ThM), *S. mitis* on Chitlac-nAg coated thermoset (AgM), *S. mitis* in the presence of saliva on Chitlac coated thermoset (ThMS), *S. mitis* in the presence of saliva on Chitlac-nAg coated thermoset (AgMS), HGFs in the presence of *S. mitis* on Chitlac coated thermoset (ThMH), HGFs in the presence of *S. mitis* on Chitlac-nAg coated thermoset (AgMH), HGFs in the presence of *S. mitis*, saliva and Chitlac coated thermoset (ThMHS), HGFs in the presence of *S. mitis*, saliva and Chitlac-nAg coated thermoset (AgMHS).

### Scanning electron microscopy (SEM) analysis of cell and bacteria

To evaluate HGFs adhesion on thermosets (coated with Chitlac or Chitlac-nAg) and cells morphology, SEM analysis was carried out. To better assess the interaction between eukaryotic, prokaryotic cells and devices, thermosets were observed with adherent HGFs, with bacteria, or with the co-culture in the presence or absence of saliva. HGFs (12.5 × 10^3^/thermoset) were seeded on each thermoset placed in a 24-well microtiter plate. Where required, thermosets were pre-treated with saliva and HGFs were allowed adhering for 3 h. After 3 h, 1 % sucrose DMEM was added. Bacteria were added when required. After incubation for 48 h in a humidified atmosphere of 5 % (v/v) CO_2_ at 37 °C, supernatants were removed and samples were gently washed with PBS and then fixed with 2.5 % glutharaldehyde in 0.1 mol/L cacodylate buffer, pH 7.6 for 20 min at 4 °C. Samples were then dehydrated in alcohol at progressively higher concentration and then in hexamethyldisazilane (Sigma Aldrich, MO, USA). The specimens were mounted onto aluminium stubs and gold sputtered in an Emitech K 550 (Emitech Ltd, Ashford, Kent, UK). Images acquired means of a SEM with LaB6 electron beam (Zeiss EVO 50 XVP, Carl Zeiss SMY Ltd, Cambridge, UK), equipped with tetra solid-state BSE detector. SEM operating conditions included 10 kV accelerating voltage, 10 mm working distance and a 1.2 nA probe current. The observations were made under variable pressure at 0.75 torr using both the BSE and SE1 detectors. The images were captured with a line average technique using 20 scans.

### Cytotoxicity (LDH) assay

HGFs were seeded at 200 000 cells/well on 24 wells plates. After 24 h, culture medium was replaced with 1 ml of DMEM 1 % sucrose. Where indicated, Chitlac and Chitlac-nAg thermosets, saliva and/or the standardized bacterial cultures were added (final volume 1 ml). After 48 h, medium was harvested and lactate dehydrogenase based assay (LDH assay, TOX-7, Sigma-Aldrich, St. Louis, MO) was performed on culture media according to manufacturer instructions. As the positive control, cells were lysed with Triton 1 %. Each test was performed in quadruplicate. Assessment of relative cytotoxicity (*CT*_*rel*_) was calculated according to the formula:

$$CT_{rel} = \frac{{\left( {\% \,LDH\,released} \right)_{sample} }}{{\left( {\% \,LDH\,released} \right)_{untreated} }} = \frac{{\left[ {{\raise0.7ex\hbox{${\left( {A - B} \right)}$} \!\mathord{\left/ {\vphantom {{\left( {A - B} \right)} {\left( {C - B} \right)}}}\right.\kern-0pt} \!\lower0.7ex\hbox{${\left( {C - B} \right)}$}}} \right] \cdot 100}}{{\left[ {{\raise0.7ex\hbox{${\left( {U - B} \right)}$} \!\mathord{\left/ {\vphantom {{\left( {U - B} \right)} {\left( {C - B} \right)}}}\right.\kern-0pt} \!\lower0.7ex\hbox{${\left( {C - B} \right)}$}}} \right] \cdot 100}} = \frac{{\left( {A - B} \right)}}{{\left( {U - B} \right)}}$$

with A = LDH activity of sample, B = LDH activity of medium, U = LDH activity of untreated HGFs and C = LDH activity of the positive control [26].

### Quantification of human collagen type 1 in culture medium (ELISA)

When fibroblasts are cultured, both pro-collagen and collagen (mature type) are secreted into culture medium. Levels of collagen in culture medium can be quantified by means of ELISA (Human collagen type 1 ELISA, Cosmobio, Tokyo, Japan). HGFs (200.000/well) were seeded into a 24-well culture plate in DMEM containing 10 % FBS, 1 % streptomycin and 1 % penicillin. After 24 h the medium was changed with 1 % sucrose DMEM. Thermosets were placed onto cell monolayers. Bacteria were added where required. After 24 and 48 h supernatants were collected and ELISA assay was carried out. Concentration of Collagen type I was calculated using a standard curve generated with specific standards provided by the manufacturers.

### Confocal microscopy analysis of cells and bacteria

#### Live/dead staining

For microscopic observations of live-dead cells, *Streptococcus mitis* alone and *Streptococcus mitis*/HGFs co-culture were incubated on thermosets for 48 h in a humidified atmosphere of 5 % (v/v) CO_2_ at 37 °C. After the incubation, the planktonic phase was removed by aspiration and each thermoset was washed gently with PBS and stained with Live/Dead Kit as indicated by the manufactures. Bacterial cells were rinsed once with PBS and stained by using a LIVE/DEAD *Bac*Light Viability kit (Molecular Probes Inc., Invitrogen, Italy). The solution (1 ml) containing SYTO 9 and propidium iodide (1 : 1) was added onto the thermosets. The samples were incubated at room temperature for 15 min in the dark. After incubation, residual stain was removed. The images were observed using a fluorescence microscope (Reichert) equipped with a halogen lamp, Neoplan 100/1.25 oil objective and 1713 filter cube (fluorescein; 490/510/520 nm).

#### Antibodies staining

To assess integrin β 1 and PKCα expression and localization in HGFs seeded on both thermosets (coated and uncoated with silver nanoparticles), immunofluorescence staining was performed. Cells (12500/thermoset) were seeded on each thermoset (where required pre-treated with saliva) and allowed to adhere for at least 3 h. Bacteria were added where required. After 48 h, cells were fixed for 10 min with PBS 1X/3 % paraformaldehyde supplemented with 2 % sucrose. Then cells membranes were permeabilized for 5 min at room temperature, with a pH 7.6 solution containing 0.5 % Triton X100/20 mM HEPES, 300 µM sucrose, 50 mM NaCl, 3 mM MgCl_2_. After membrane permeabilization, cells were incubated with PBS 1X/BSA 10 % for 30 min at room temperature, followed by 4 h incubation at 37 °C with anti-integrin β1 mouse monoclonal antibody (Santa Cruz Biotechnology, CA, USA) 1:20 in 1 % BSA/PBS, and with anti-PKCα rabbit monoclonal antibody (Abcam, Cambridge, UK) 1:100 in 1 % BSA/PBS. Alexa-568 goat anti-mouse and Alexa-488 goat anti rabbit (both 1:50 in 1 % BSA/PBS) secondary antibodies were added and samples were incubated for 2 h at 37 °C.

#### Confocal microscopy analysis

Images were acquired using a confocal microscope (LSM510 META, Carl Zeiss, Germany), equipped with an inverted microscope (Axio Observer) and an EC-Plan Neofluar 63X NA 1.25 and 100X NA 1.3 OIL lens. The excitation was obtained with an Argon laser line (488 nm) and a HeNe laser line (543 nm). The emission was collected through a primary dichroic Filter (HTF) 488/543 nm and separate with a secondary dichroic filter NTF545. In Integrin (Green)/PKC (Red) and Live (Green)/death (Red) staining, emission was recorded selecting a BP 505–530 nm for Green and BP 585–615 nm for Red. To prevent fluorescence emissions overlaps, images were acquired sequentially (Multitrack mode).

Ten fields, for each slide randomly chosen were examined. Densitometry and the counts of viable adhering bacteria on thermoset or on HGF were carried out by using image analysis software (LEICA QWin, Germany). Microscopic observations were repeated for three independent experiments.

### Statistics

Statistical analysis was performed using the *t*-student test. Results are expressed as the mean ± SD. Values of *P* < 0.05 were considered statistically significant.

## Results

### Confocal 3D surface rendering and SEM observation and quantification

**Fig. 1:**
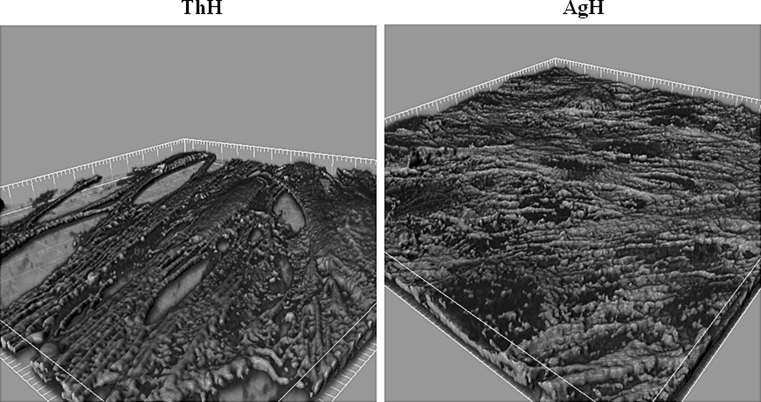
3D surface rendering of β1 integrin with confocal microscopy. Images show HGFs cultured onto Chitlac (ThH) and Chitlac-nAg (AgH) thermosets. HGFs were stained for β1 integrin. Magnification ×400

**Fig. 2:**
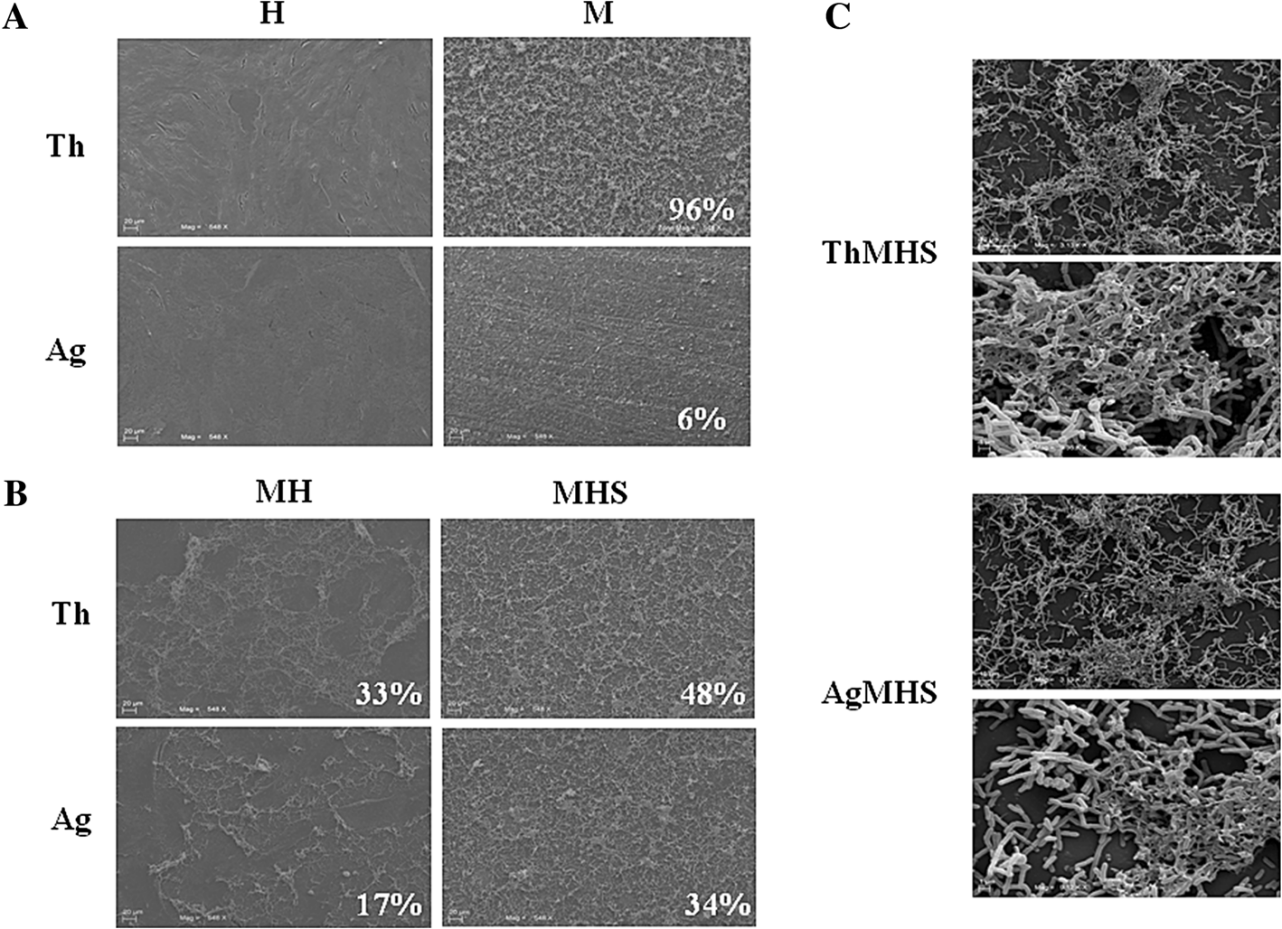
Scanning electron microscopy (SEM) on HGFs and HGFs/*S. mitis* co-culture in the presence of Chitlac and Chitlac-nAg thermosets. **a** Representative images of HGFs (H), *S. mitis* (M) and **b** HGFs in the presence of *S. mitis* and/or saliva (MH, MHS) cultured on Chitlac (Th) and Chitlac-nAg (Ag) thermosets (magnification ×548). *Percentages* indicate the densitometries of bacteria observed onto thermosets. **c** Representative images of *S. mitis* biofilm on Chitlac (ThMHS) and Chitlac-nAg thermosets (AgMHS). Magnification ×31300 (**a**, **c**), ×93600 (**b**, **d**). For abbreviation see Sect. 2

Figure 1 shows the 3D surface rendering of HGFs growth onto Chitlac and Chitlac-nAg thermosets stained for β1 integrin. These images allow to visualize HGFs monolayer formed on both surfaces. In order to assess the ability of HGFs, *S. mitis* and the co-culture to adhere onto BisGMA/TEGDMA thermosets, SEM observations and quantifications were run out. Figure 2a shows SEM images of the different two surfaces with HGFs or *S. mitis*. These images show that cells uniformly proliferate creating a monolayer which adheres onto the substrate, both in presence with Chitlac-nAg (AgH) or Chitlac alone (ThH). As regard *S. mitis*, the percentage of cells growing onto Chitlac thermosets is significantly higher (96 % of the total surface analysed) with respect to those growing on Chitlac-nAg (6 %). Figure 2b exhibits HGFs co-cultured with *S*. *mitis* growing onto thermosets. It should be noted that bacteria percentage decreased in the presence of AgNPs (ThMH 33.06 %; AgMH 17.3 %), while when saliva is added, a greater bacterial aggregation onto the surfaces is shown (ThMHS 48.34 %, AgMHS 33.74 %). In Fig. 2c the sessile microbial proliferation with cells embedded in the extracellular matrix formation is shown.

### Live/dead staining

**Fig. 3:**
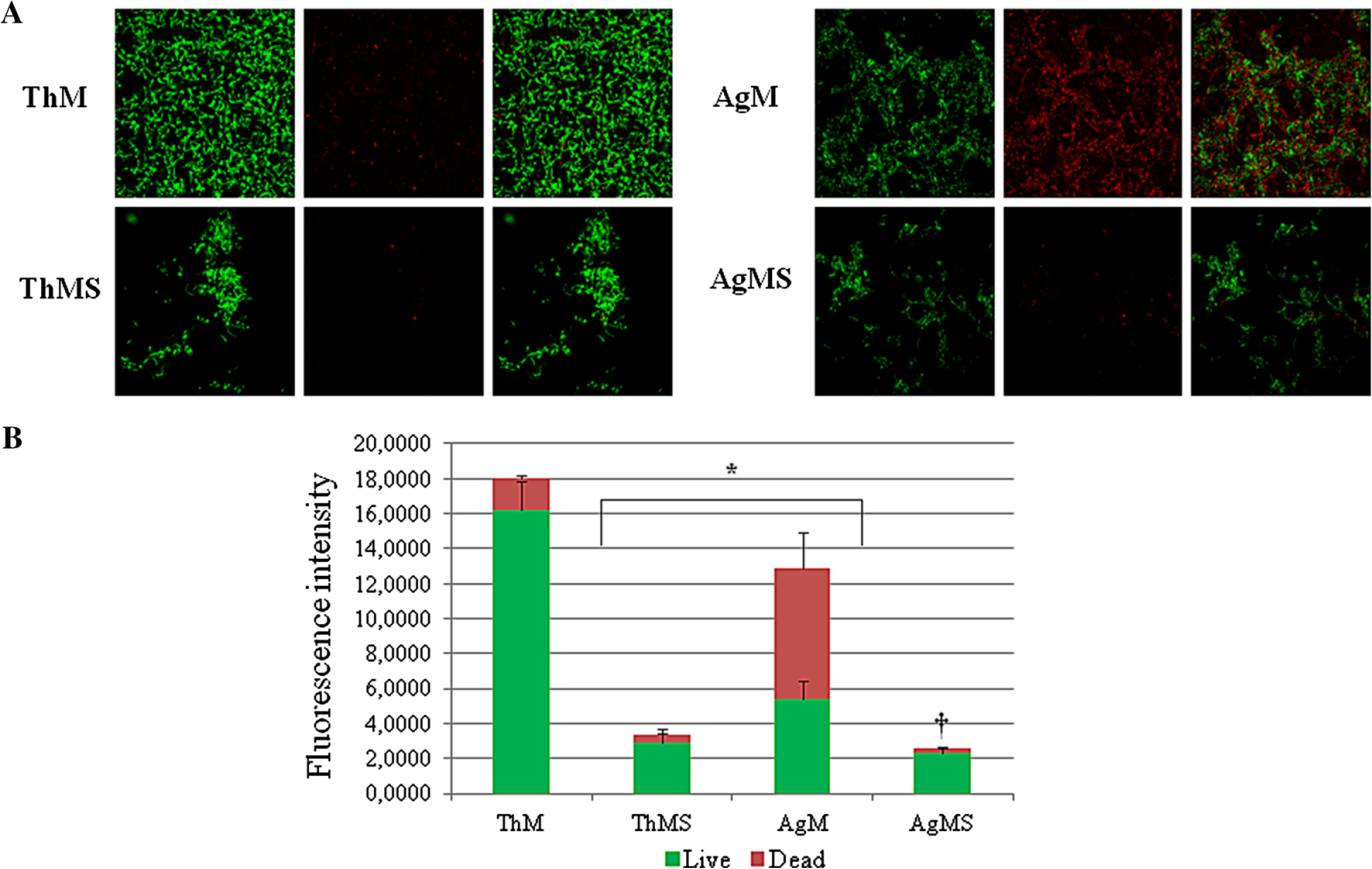
Live/Dead staining of *S. mitis* cultured in the presence of Chitlac and Chitlac-nAg thermosets. **a** Representative confocal images of *S. mitis* cultured for 48 h in the presence of Chitlac and Chitlac-nAg thermosets stained with Live/Dead. Green stained bacteria represent the viable populations. Magnification ×400. **b** Statistical histogram representation of Live/Dead staining. * AgM and ThMS vs ThM *P* < 0.05; † AgMS vs AgM *P* < 0.05. For abbreviation see Sect. 2

Live/Dead assay was performed and the fluorescence intensity (FI) of the staining was measured on bacteria growing onto Chitlac and Chitlac-nAg BisGMA/TEGDMA thermosets, in order to visualize and quantify the viability and the proliferation rate of *S. mitis* in the different experimental situations (Fig. 3a, b). This analysis confirms SEM quantification. An enormous amount of living cells is evidenced onto Chitlac thermosets (FI_live_ = 16.18; FI_dead_ = 1.8), while these values decrease when *S*. *mitis* is cultured onto thermosets coated with Chitlac-nAg (FI_live_ = 4.68; FI_dead_ = 6.38) (Fig. 3b). When saliva is added to the culture, the amount of detectable cells through Live/Dead staining decreases, both in Chitlac and Chitlac-nAg thermosets. In Fig. 3a, images referred to ThMS and AgMS show live adherent bacterial aggregates (biofilm) stained in green. Due to bacterial aggregation, the staining overlaps and therefore a lower FI values are detected. (FI_live_ = 2.92 and 2.29, respectively).

### LDH cytotoxicity assay

**Fig. 4:**
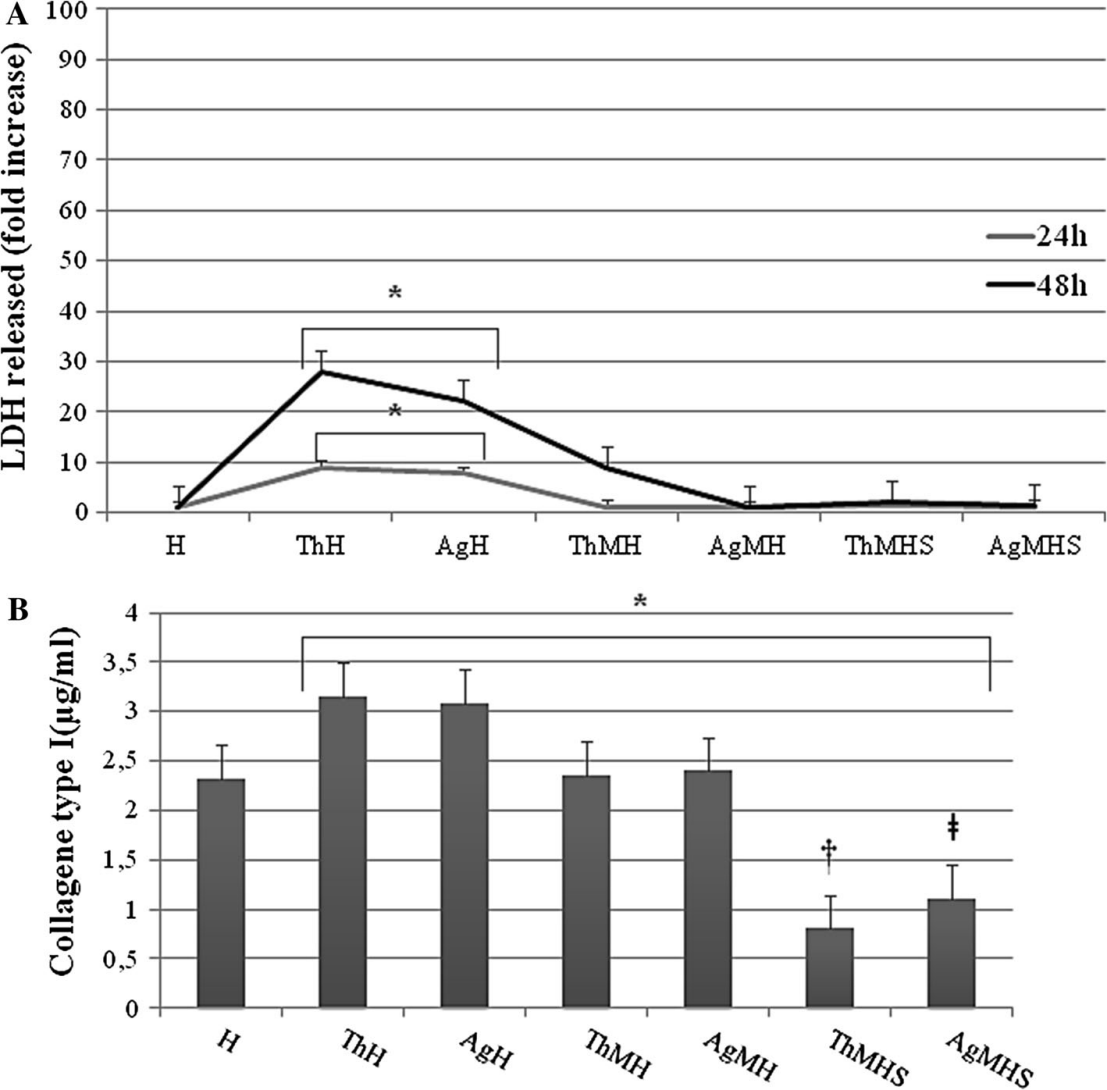
Lactate dehydrogenase (LDH) and human collagen type I release in HGFs cultured in the presence of Chitlac and Chitlac-nAg thermosets. **a** Graph represents LDH release (fold increase) in HGFs cultured at different conditions on Chitlac (Th) and Chitlac-nAg (Ag) thermosets after 24 and 48 h. * ThH and AgH vs H 24 and 48 h *P* < 0.05. **b** Statistical histogram represents human collagen type I release (µg/ml) in HGFs cultured in the presence of Chitlac (Th) and Chitlac-nAg (Ag) thermosets after 48 h. ThH, AgH, ThMH, AgMH, ThMHS and AgMHS vs H *P* < 0.05; † ThMHS vs ThH and ThMH *P* < 0.05; ǂ AgMHS vs AgH and AgMH *P* < 0.05. For abbreviation see Sect. 2

Chitlac and Chitlac-nAg exert only a minimal cytotoxic effect on HGFs at 24 h (8.9 and 7.8 fold higher, respectively), while LDH release at 48 h rises up to 27.9 and 22.0 (Fig. 4a). Moreover, in the presence of *S. mitis*, a greater reduction of LDH release by HGFs is detected in the presence of Chitlac-nAg coated thermosets (indicated in figure as AgMH, 1.0 fold) with Chitlac alone (indicated as ThMH, 9.0 fold) after 48 h. The addition of saliva in the culture medium even more reduces LDH release after 48 h (1.9 fold Chitlac thermoset, 1.4 fold Chitlac-nAg thermoset) (Fig. 4a).

### Quantification of human collagen type 1 in culture media (ELISA assay)

In order to investigate the amount of collagen secreted into the culture medium by HGFs alone or in co-culture with *S*. *mitis* in the presence of BisGMA/TEGDMA thermosets, ELISA assay was performed (Fig. 4b). After 48 h of treatment, collagen secretion rises in the presence of Chitlac coated thermosets with respect to HGFs alone (3.157 μg/ml vs 2.318 μg/ml), as well as in the presence of Chitlac-nAg thermosets (3.09 μg/ml) The co-culture system shows a decrease in collagen production dramatically evident in the presence of saliva (0.806 μg/ml ThMHS; 1.104 μg/ml AgMHS).

### Confocal microscopy of integrin β1 and protein kinase Cα (PKC α)

**Fig. 5:**
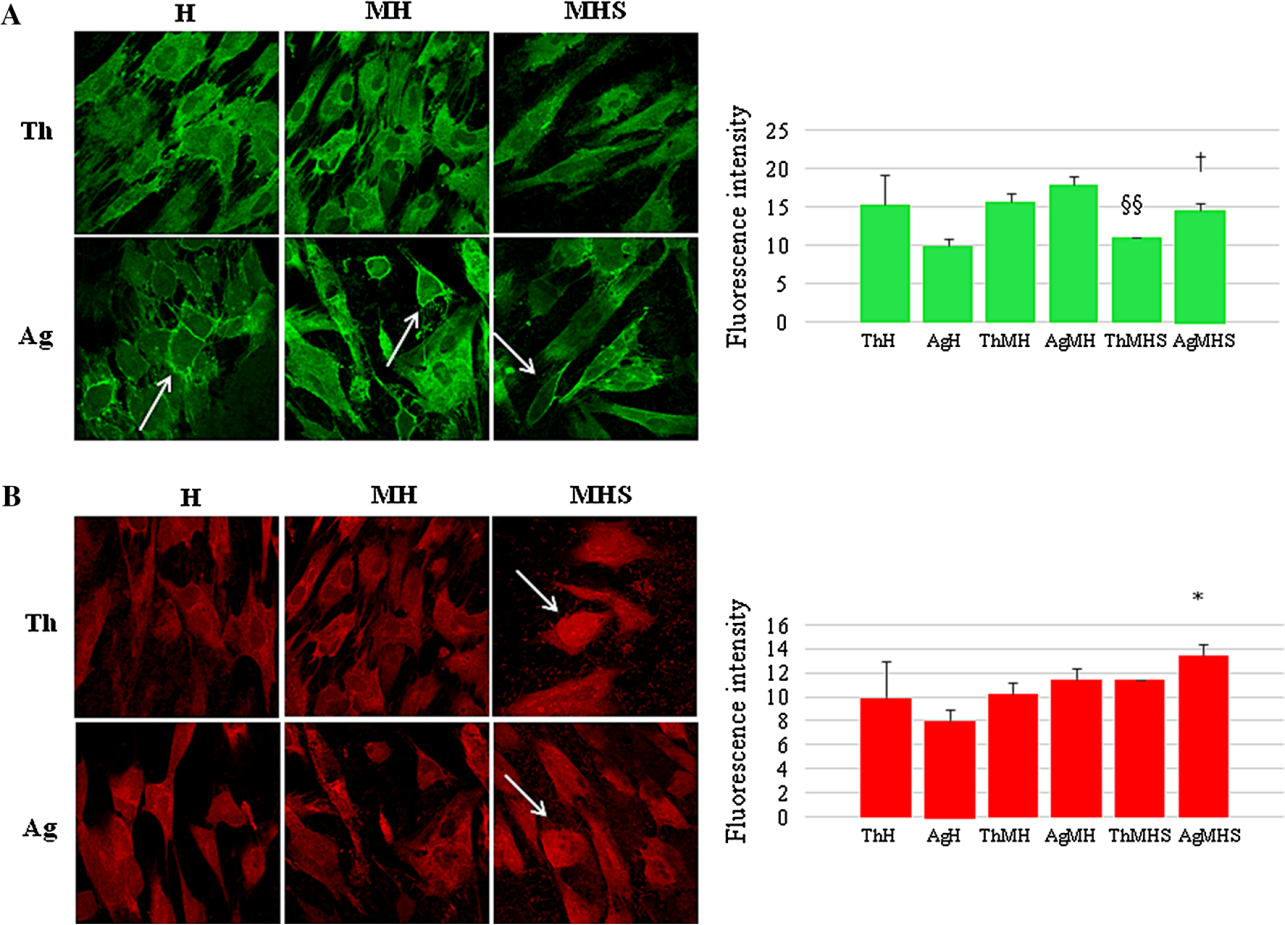
**a** Integrin β1 expression in HGFs cultured onto Chitlac and Chitlac-nAg thermosets after 48 h. Representative confocal images of HGFs stained for β1 integrin (magnification ×400). *Arrows* indicate integrin β1 cell membrane localization. Graph outlines the statistical quantitative expression of β1 integrin.§§ AgMH vs AgH *P* < 0.05; † AgMHS vs AgMH *P* < 0.05. **b** PKC α expression in HGFs cultured onto Chitlac and Chitlac-nAg thermosets after 48 h. Representative confocal images of HGFs stained for PKC α (magnification ×400). *Arrows* indicate PKC α nuclear localization. Graph outlines the statistical quantitative expression of PKC α. * AgMHS vs AgH *P* < 0.05

Immunofluorescence staining images and quantification of the fluorescence intensity (FI) of integrin β1 expression are shown in Fig. 5a. As regard Chitlac thermosets, in the presence of *S. mitis* and saliva the amount of protein does not undergo any modifications. When HGFs are co-cultured with *S. mitis* onto Chitlac-nAg thermosets, the expression of total β1 integrin increases (AgMH FI = 17.94 vs AgH FI = 9.89) as well as when saliva is added (FI = 14.53). White arrows show integrin β1 localized closely to cell membranes in HGFs cultured onto Chitlac-nAg thermosets.

In Fig. 5b, HGFs are stained for PKC α and analyzed through a confocal microscope. In the panel on the left, representative images of HGFs in the different experimental conditions are shown. While the amount of total protein does not undergo significant modifications, with the exception of AgMHS (FI = 13.39 vs 7.95), images show nuclear translocation of PKC α when HGFs are co-cultured with *S. mitis* onto both thermosets (coated with Chitlac or Chitlac-nAg) in the presence of saliva (ThMHS and AgMHS).

## Discussion

Dental implant materials interact with several cell types, including osteoblasts, periodontal ligament fibroblasts, monocytes and human gingival fibroblasts [27]. Implant-associated infections are the major complications in dental surgery which could lead to implant failure. To date dental research is focused on silver nanoparticle technology for the well-known antimicrobial properties and the resulting findings are encouraging [6]. However, there are controversial results with respect to the possible adverse effects and toxicity of silver nanoparticles for eukaryotic cells [28]. Several reports focused on the ability of positively charged polyelectrolytes, chitosan for instance, to stimulate cell adhesion [26, 29]. In our experimental model, coating thermosets with Chitlac and Chitlac-nAg allows HGFs forming a monolayer on both surfaces (Figs. 1, 2). SEM observations confirm the good adhesion of HGFs on both thermosets (Fig. 2a). In order to test the antimicrobial activity of our devices we used a commensal bacterial strain colonizing the oral cavity, *S. mitis*. Microscopic images show that in the presence of silver nanoparticles the amount of viable bacteria dramatically decrease (Fig. 2a). In order to mimic interactions occurring between eukaryotic/prokaryotic cells and composites, HGFs were co-cultured with *S. mitis* in the presence of saliva as previously described [25]. When co-cultured, the presence of silver nanoparticles does not affect HGFs adherence on thermosets, while the amount of viable Streptococci decreases even more in the presence of saliva. To protect themselves from a hostile environment, bacteria often form surface-attached communities described as bacterial biofilms. Biofilms are ubiquitous in natural, industrial, and clinical environments and have been shown to play a critical role in many chronic infections. Biofilms are usually composed of multiple bacterial species. For example, dental biofilms (i.e., dental plaque) contain more than 500 different bacterial species. Biofilms consist of viable microbial cells along with dead cells and a wide range of self-generated extracellular polymeric substances (EPS) including polysaccharides, nucleic acids (extracellular DNA from bacteria), and proteins [30]. The increased aggregation of *S. mitis* forming biofilm with saliva is confirmed by SEM observation in Fig. 2c. In order to evaluate the antimicrobial activity of Chitlac-nAg coated thermosets, Live/Dead staining was performed (Fig. 3). This confirms SEM observations with the lowest amount of viable bacterial cells observed on thermosets coated with silver nanoparticles. The staining overlaps exactly when saliva is added showing biofilm formation. Although the antibacterial mechanism is not totally understood, the main molecular targets of silver nanoparticles are supposed to be the thiol groups (–SH) of proteins exposed to the extracellular portion of the bacterial membrane; conversely, eukaryotic cells lack these exterior binding sites, so the nanoparticles are supposed to interact with them only upon metal internalization. The lack of physical barriers to nanoparticle diffusion into cells represents a risk of uptake by eukaryotic cells, which can lead to their death [10, 11]. LDH release after 24 and 48 h increases on both surface when HGFs are cultivated alone (Fig. 4a). In the presence of *S. mitis* the amount of LDH decreases even more when saliva is added, confirming a protective role of bacteria on HGFs as previously described [25, 31]. Collagen type I is the principal biopolymer in the extracellular matrix of both vertebrates and invertebrates. It is prevalent in connective tissues, and the arrangement of collagen determines the mechanical response and the adhesion properties [32]. HGFs collagen type I secretion is not affected by the presence of Chitlac and Chitlac-nAg thermosets, being even more increased when they are cultured on both surfaces, confirming again SEM observations (Fig. 4b). When HGFs are co-cultured with *S. mitis*, the amount of collagen is comparable with the amount in control sample, while the presence of saliva decreases the secretion of collagen type I. Previous studies demonstrated that oral tissues, such as cementum, root, and periodontal ligament fibers, rich in collagen type I, if exposed to the oral environment, may be vulnerable to attachment and colonization by organisms equipped with collagen-binding adhesions, leading to the formation of biofilm [33]. In addition to specific adhesions, the presence of immobilized fibril-forming collagens could promote the formation of dense biofilm. The induction of a dense biofilm is also observed in the presence of collagen type I in solution [34]. It is plausible to assume that the decreased collagen secretion in the supernatant detected by ELISA assay is due to the biofilm formation and the consequent digestion of collagen type I by *S. mitis*.

Gingival fibroblasts produce type I collagen which represents a scaffold for numerous associated proteins in the extracellular matrix, but also produce integrin β1, a protein involved in the regulation of cell migration, proliferation, survival/apoptosis and differentiation. Among the intracellular signaling pathways activated by integrins, PKC isoenzymes are included [23, 31]. Chitlac coated thermosets (Th) do not particularly affect the amount of total expression of integrin β1, confirming that HGFs adhere well on these surfaces (Fig. 5a). In the presence of Chitlac-nAg thermosets, the amount of integrin β1 increases and more important, it appears localized closely to the cell membrane, confirming that HGFs, notwithstanding the presence of silver nanoparticles which could disturb cell attachment, adhere well on Chitlac-nAg thermosets (Fig. 5a, white arrows). In parallel, a PKC α activation as nuclear translocation can be visualized in Fig. 5b indicated by white arrows.

Based on the above considerations, a key role of the oral environment in preventing cellular toxic events occuring in human gingival fibroblasts can be highlighted. Moreover cells are well attached on Chitlac and Chitlac-nAg and the amount of bacteria decreases in the presence of silver nanoparticles, confirming the antimicrobial properties of this coating and its biocompatibility. In conclusion, the nanocomposite layer formed by the polysaccharide Chitlac and Chitlac-nAg has a considerable potential as a good performing surface coating for biomaterials usable in restorative dentistry.

## References

[1] Yue C, Zhao B, Ren Y, Kuijer R, van der Mei HC, Busscher HJ, Rochford ET (2015). The implant infection paradox: why do some succeed when others fail? Opinion and discussion paper. Eur Cell Mater.

[2] Belibasakis GN, Charalampakis G, Bostanci N, Stadlinger B (2015). Peri-implant infections of oral biofilm etiology. Adv Exp Med Biol.

[3] Eberhard J, Pietschmann R, Falk W, Jepsen S, Dommisch H (2009). The immune response of oral epithelial cells induced by single-species and complex naturally formed biofilms. Oral Microbiol Immunol.

[4] Nascimento CD, Pita MS, Fernandes FH, Pedrazzi V, de Albuquerque Junior RF, Ribeiro RF (2014). Bacterial adhesion on the titanium and zirconia abutment surfaces. Clin Oral Implants Res.

[5] Jhajharia K, Parolia A, Shetty KV, Mehta LK (2015). Biofilm in endodontics: a review. J Int Soc Prev Community Dent.

[6] Brennan SA, Ní Fhoghlú C, Devitt BM, O’Mahony FJ, Brabazon D, Walsh A (2015). Silver nanoparticles and their orthopaedic applications. Bone Joint J.

[7] Zhang Y, Zheng Y, Li Y, Wang L, Bai Y, Zhao Q, Xiong X, Cheng Y, Tang Z, Deng Y, Wei S (2015). Tantalum nitride-decorated titanium with enhanced resistance to microbiologically induced corrosion and mechanical property for dental application. PLoS One.

[8] Travan A, Pelillo C, Donati I, Marsich E, Benincasa M, Scarpa T, Semeraro S, Turco G, Gennaro R, Paoletti S (2009). Non-cytotoxic silver nanoparticle-polysaccharide nanocomposites with antimicrobial activity. Biomacromolecules.

[9] Piao MJ, Kang KA, Lee IK, Kim HS, Kim S, Choi JY, Choi J, Hyun JW (2011). Silver nanoparticles induce oxidative cell damage in human liver cells through inhibition of reduced glutathione and induction of mitochondria-involved apoptosis. Toxicol Lett.

[10] Marsich E, Travan A, Donati I, Turco G, Kulkova J, Moritz N, Aro HT, Crosera M, Paoletti S (2013). Biological responses of Silver-coated thermosets: an in vivo and in vitro study. Acta Biomater.

[11] Travan A, Marsich E, Donati I, Benincasa M, Giazzon M, Felisari L, Paoletti S (2011). Silver-polysaccharide nanocomposite antimicrobial coatings for methacrylic thermosets. Acta Biomater.

[12] Besinis A, De Peralta T, Handy RD (2014). The antibacterial effects of silver, titanium dioxide and silica dioxide nanoparticles compared to the dental disinfectant chlorhexidine on Streptococcus mutans using a suite of bioassays. Nanotoxicology.

[13] Schuster L, Rothmund L, He X, Van Landuyt KL, Schweikl H, Hellwig E, Carell T, Hickel R, Reichl FX, Högg C (2015). Effect of Opalescence(^®^) bleaching gels on the elution of dental composite components. Dent Mater.

[14] Carpenter GH (2013). The secretion, components, and properties of saliva. Annu Rev Food Sci Technol.

[15] Schweikl H, Hiller KA, Carl U, Schweiger R, Eidt A, Ruhl S, Müller R, Schmalz G (2013). Salivary protein adsorption and Streptococccus gordonii adhesion to dental material surfaces. Dent Mater.

[16] Inui T, Walker LC, Dodds MW, Hanley AB (2015). Extracellular glycoside hydrolase activities in the human oral cavity. Appl Environ Microbiol.

[17] Di Giulio M, D’Ercole S, Zara S, Cataldi A, Cellini L (2011). *Streptococcus mitis*/human gingival fibroblast co-culture: the best natural association in answer to the 2-hydroxyethyl methacrilate release. APMIS.

[18] Mitchell J (2011). *Streptococcus mitis*: walking the line between commensalism and pathogenesis. Mol Oral Microbiol.

[19] Zara S, Di Giulio M, D’Ercole S, Cellini L, Cataldi A (2011). Anti-adhesive and pro-apoptotic effects of 2-hydroxyethyl methacrylate on human gingival fibroblast co-cultured with *Streptococcus mitis* strains. Int Endod J.

[20] Di Giulio M, di Giacomo V, Di Campli E, Di Bartolomeo S, Zara S, Pasquantonio G, Cataldi A, Cellini L (2013). Saliva improves *Streptococcus mitis* protective effect on human gingival fibroblasts in presence of 2-hydroxyethyl-methacrylate. J Mater Sci Mater Med.

[21] Elloumi-Hannachi I, García JR, Shekeran A, García AJ (2015). Contributions of the integrin β1 tail to cell adhesive forces. Exp Cell Res.

[22] Di Nisio C, De Colli M, di Giacomo V, Rapino M, Di Valerio V, Marconi GD, Gallorini M, Di Giulio M, Cataldi A, Zara S (2015). A dual role for β1 integrin in an in vitro *Streptococcus mitis*/human gingival fibroblasts co-culture model in response to TEGDMA. Int Endod J.

[23] Cataldi A, Zara S, Rapino M, Patruno A, di Giacomo V (2013). Human gingival fibroblasts stress response to HEMA: a role for protein kinase C α. J Biomed Mater Res A.

[24] Yalpani M, Hall LD (1984). Some chemical and analytical aspects of polysaccharide modifications. Formation of branched-chain, soluble chitosan derivatives. Macromolecules.

[25] Gallorini M, Sancilio S, Zara S, De Colli M, Di Giulio M, Cataldi A, di Giacomo V (2014). Involvement of mitochondrial signalling pathway in HGFs/S. mitis coculture response to TEGDMA treatment. J Biomed Mater Res A.

[26] Sancilio S, di Giacomo V, Di Giulio M, Gallorini M, Marsich E, Travan A, Tarusha L, Cellini L, Cataldi A (2014). Biological responses of human gingival fibroblasts (HGFs) in an innovative co-culture model with *Streptococcus mitis* to thermosets coated with a silver polysaccharide antimicrobial system. PLoS One.

[27] Pivodova V, Frankova J, Ulrichova J (2011). Osteoblast and gingival fibroblast markers in dental implant studies. Biomed Pap Med Fac Univ Palacky Olomouc Czech Repub.

[28] Nganga S, Travan A, Marsich E, Donati I, Söderling E, Moritz N, Paoletti S, Vallittu PK (2013). In vitro antimicrobial properties of silver-polysaccharide coatings on porous fiber-reinforced composites for bone implants. J Mater Sci Mater Med.

[29] Travan A, Marsich E, Donati I, Foulc MP, Moritz N, Aro HT, Paoletti S (2012). Polysaccharide-coated thermosets for orthopedic applications: from material characterization to in vivo tests. Biomacromolecules.

[30] Jefferson KK (2004). What drives bacteria to produce a biofilm?. FEMS Microbiol Lett.

[31] di Giacomo V, Pacella S, Rapino M, Di Giulio M, Zara S, Pasquantonio G, Cellini L, Cataldi A (2013). pPKC α regulates through integrin β 1 human gingival fibroblasts/*Streptococcus mitis* adhesion in response to HEMA. Int Endod J.

[32] Sherman VR, Yang W, Meyers MA (2015). The materials science of collagen. J Mech Behav Biomed Mater.

[33] Miller JH, Avilés-Reyes A, Scott-Anne K, Gregoire S, Watson GE, Sampson E, Progulske-Fox A, Koo H, Bowen WH, Lemos JA, Abranches J (2015). The collagen binding protein Cnm contributes to oral colonization and cariogenicity of Streptococcus mutans OMZ175. Infect Immun.

[34] Chagnot C, Agus A, Renier S, Peyrin F, Talon R, Astruc T, Desvaux M (2013). In vitro colonization of the muscle extracellular matrix components by Escherichia coli O157:H7: the influence of growth medium, temperature and pH on initial adhesion and induction of biofilm formation by collagens I and III. PLoS One.

